# Changes in the Bronchial Cuff Pressure of Left-Sided Double-Lumen Endotracheal Tube by Lateral Positioning: A Prospective Observational Study

**DOI:** 10.3390/jcm10081590

**Published:** 2021-04-09

**Authors:** Jong-Hae Kim, Eugene Kim, In-Young Kim, Eun-Joo Choi, Sung-Hye Byun

**Affiliations:** 1 Department of Anesthesiology and Pain Medicine, School of Medicine, Daegu Catholic University, 33, Duryugongwon-ro 17-gil, Nam-gu, Daegu 42472, Korea; usmed12@gmail.com (J.-H.K.); kiy215@naver.com (I.-Y.K.); sthgood9@naver.com (E.-J.C.); 2 Department of Anesthesiology and Pain Medicine, Hanyang University Medical Center, College of Medicine, Hanyang University, 222-1, Wangsimni-ro, Seongdong-gu, Seoul 04763, Korea; tomomie@hanmail.net; 3 Department of Anesthesiology and Pain Medicine, Kyungpook National University Chilgok Hospital, School of Medicine, Kyungpook National University, 807, Hoguk-ro, Buk-gu, Daegu 41404, Korea

**Keywords:** double-lumen tube, lateral position, cuff, pressure, thoracic surgery

## Abstract

Proper bronchial cuff pressure (BCP) is important when using a double-lumen endotracheal tube (DLT), especially in thoracic surgery. As positional change during endotracheal tube placement could alter cuff pressure, we aim to evaluate the change in BCP of DLT from the supine to the lateral decubitus position during thoracic surgery. A total of 69 patients aged 18–70 years who underwent elective lung surgery were recruited. BCP was measured at a series of time points in the supine and lateral decubitus positions after confirming the DLT placement. The primary outcome was change in the initial established BCP (BCPi), which is the maximum pressure at which the BCP did not exceed 40 cmH_2_O without air leak in the supine position, after lateral decubitus positioning. As the primary outcome, the BCPi increased from 25.4 ± 9.0 cmH_2_O in the supine position to 29.1 ± 12.2 cmH_2_O in the lateral decubitus position (*p* < 0.001). Out of the 69 participants, 43 and 26 patients underwent surgery in the left-lateral decubitus position (LLD group) and the right-lateral decubitus position (RLD group) respectively. In the LLD group, the BCPi increased significantly (*p* < 0.001) after lateral positioning and the beginning of surgery and the difference value, ∆BCPi, from supine to lateral position was significantly higher in the LLD group than in the RLD group (*p* = 0.034). Positional change from supine to lateral decubitus could increase the BCPi of DLT and the increase was significantly greater in LLD that in RLD.

## 1. Introduction

Double-lumen endotracheal tubes (DLTs) are commonly used to create adequate one-lung ventilation (OLV) in thoracic surgery. The bronchial cuff of the DLT should be not only placed in the proper position but also inflated to an appropriate pressure, in order to achieve the perfect lung isolation [1]. Inflation of the bronchial cuff with insufficient pressure can lead to air leakage due to underinflation, thereby interfering with the surgeon’s surgical field of view [2], especially in video-assisted thoracoscopic surgery (VATS). However, excessive pressure on the cuff of the DLT should be avoided to prevent cuff-related complications such as airway trauma [1], similar to those which can occur with single-lumen endotracheal tubes (ETT) [3,4,5,6,7]. Intubation using DLT has been reported to cause more airway injuries due to its stiffer characteristics and larger external diameter compared to single-lumen ETT [8]. In particular, bronchial injuries, such as mucosal edema, have been reported in studies related to DLT [9]; therefore, it is necessary for clinicians to pay attention to the bronchial cuff pressure of the DLT in order to prevent the cuff-related complication. Although there is only limited literature on the proper range of bronchial cuff pressure (BCP), the initial BCP that can be used to create an effective seal to avoid air leakage around the cuff, while avoiding bronchial damage has been thought to be 35 cmH_2_O [10,11,12].

Several studies have shown that changes in cuff pressure of ETT can occur in response to patient’s postural changes, such as movement from supine to prone [13] or lateral frank position [14] and head rotation [15,16]. Alteration in the cuff pressure has been primarily attributed to the displacement of the tube within the trachea [14]. In addition, gravity-related morphological and conformational changes of the trachea in response to body positioning can also produce a change in cuff pressure [3] Attention should be paid to changes in cuff pressure from the initially set cuff pressure in surgeries requiring a positional change. However, in the above-mentioned studies, single-lumen ETT were used and, until now, no study has been done on the BCP of DLT.

We hypothesized that a change from supine to lateral decubitus position, which is essential for thoracic surgery, would affect the BCP of DLT, even when the tube displacement problem had been eliminated through fiberoptic confirmation that the bronchial cuff was correctly positioned. The purpose of this study was to evaluate the BCP of DLT before and after a shift to the lateral decubitus position in patients undergoing thoracic surgery.

## 2. Materials and Methods

This prospective, single-center observational study was approved by the Institutional Review Board of Daegu Catholic University Medical Center (CR-18-111) on 22 August 2018. The protocol was registered at ClinicalTrials.gov (NCT03656406) and published [17]. This study was conducted at the tertiary university hospital in Daegu, South Korea, from September 2018 to April 2019 and adhered to the Strengthening the Reporting of Observational Studies in Epidemiology (STROBE) statement.

### 2.1. Participants

Patients aged 18–70 years and with an ASA (American Society of Anesthesiologists) physical status of 1 or 2 scheduled to undergo elective lung surgery requiring lateral decubitus positioning and one-lung ventilation (OLV) using a left-sided DLT were enrolled. Written informed consent was obtained from each study participant at the time of their preoperative visit.

The exclusion criteria were as follows: need for a right-sided DLT; an intraluminal lesion in the left main bronchus (LMB); an anatomical problem in the tracheobronchial tree; lung impairment, such as chronic obstructive pulmonary disease; and refusal to participate in the study.

### 2.2. Anesthesia and DLT Intubation

Standard monitoring, including electrocardiography, non-invasive blood pressure measurement and pulse oximetry, was performed after arrival in the operating room in all participants. A disposable bispectral index sensor (BIS™, Aspect Medical Systems, Newton, MA, USA) was applied to monitor the depth of anesthesia. Anesthesia was induced with propofol and remifentanil using target-controlled infusion based on bispectral index monitoring of depth of anesthesia and rocuronium 0.8 mg/kg, as a muscle relaxant, was administered to facilitate tracheal intubation. A disposable left-sided polyvinyl chloride DLT (Broncho-Cath^®^, Mallinckrodt Medical Ltd., Athlone, Ireland) was used for OLV. The size of the DLT was chosen according to the diameter of the patient’s LMB as measured on computed tomography of the chest [2]. The DLT was intubated under direct laryngoscopy and its accurate placement was confirmed by fiberoptic bronchoscopy (FOB; Olympus Optical Co., Tokyo, Japan) using the method described by Campos et al. [18]. Next, the blue-colored bronchial cuff was positioned just below the carina without herniation. While inflating the DLT cuff, the tube was fixed temporarily at the patient’s mouth using tape.

### 2.3. Measurement of Outcomes

The cuff pressure of the DLT was measured using a cuff manometer (VBM Medizintechnik GmbH™, Sulz, Germany) connected to a three-way stopcock to inject a certain amount of air and to the pilot balloon valve of the cuff. Identical manometer assembly was used in all participants. The measurements were taken 2 min after confirmation of DLT placement in each patient position. Before injection of air into the bronchial cuff via the three-way stopcock, the intracuff pressure was equilibrated at atmospheric pressure, to maintain the resting volume of the cuff, by keeping the three-way stopcock open to the outside. The BCP was assessed while inflating the cuff with air in 0.5 mL increments from 0 mL to 3.0 mL under the condition of OLV by clamping the lumen of operative side of the DLT. If the pressure had a range of values, the average of the maximum and minimum values was calculated and considered as the BCP. If the BCP exceeded 40 cmH_2_O during expansion in 0.5 mL increments from 0 mL to 3.0 mL, inflation of the bronchial cuff with air was stopped and the bronchial cuff volume (BCV) and BCP were measured up to the last numerical value. Studies have reported a varying range from 30 cmH_2_O to 44 cmH_2_O as acceptable values for the upper limit of BCP for DLT [10,11,12]. Therefore, 40 cmH_2_O was set as the upper limit of BCP in this study. Air leak around the bronchial cuff was also checked at each time point while inflating the cuff in 0.5 mL increments. In the present study, simple methods including capnography, pressure-volume loop present on the respiratory monitor of the anesthetic machine, or measurement of the exhaled return tidal volume (TVe) were used to detect the air leak. If the configuration on capnography or the pressure-volume loop was distorted or TVe was less than 80% of the established tidal volume (TVset), an air leak was considered to be present.

The maximum pressure at which the BCP did not exceed 40 cmH_2_O without air leak and the volume at that time point were defined as the maximum BCP (BCPmax) and maximum BCV, respectively and as the initial established BCP (BCPi) and initial established BCV (BCVi), respectively, if in the supine position. The smallest BCV without air leak and the pressure at that time point were defined as minimum BCV and minimum BCP, respectively. The tracheal cuff pressure was modified using a cuff manometer to maintain pressure in the range of 20–30 cmH_2_O and the volume of air injected was measured. Ventilation was maintained at a tidal volume of 6–8 mL/kg body weight and the respiration rate was adjusted to maintain an end-tidal carbon dioxide value of 30–35 mmHg and a positive end-expiratory pressure (PEEP) of 5 cmH_2_O. The ventilation mode and setting were kept constant in both patient positions during OLV to observe the effect of positional change on peak inspiratory pressure (PIP), which could affect the cuff pressure. After measuring these values, the bronchial cuff was aspirated completely and equilibrated at atmospheric pressure, thereby returning to its resting state under two-lung ventilation. The length of the LMB was also measured before and after lateral positioning to check for any conformational change. As described in our previous report [19], when the tip of the FOB reached the tracheal or bronchial carina, the point of contact with the elbow connector of the DLT was marked on the tape previously attached to the shaft of the FOB and the length between these two marks was taken as the length of the LMB. All the FOB procedures, including confirmation of the correct position of the DLT and measurement of length, were performed by two experienced investigators (E.K., S.H.B.) with at least 5 years of experience in thoracic anesthesia.

After the patient was placed in the lateral decubitus position with an axillary roll under the dependent axilla, the operating table was flexed enough to allow the shoulder and hip to be placed on the horizontal line and the intercostal space to be widened maximally. All the positioning procedures were undertaken by an independent senior resident. The bronchial cuff was maintained at the same position before and after moving the patient into the lateral position by the same investigator and the length of the LMB was measured using another FOB. After inflating the tracheal cuff using the administered volume of air in the supine position, the cuff pressure was checked using a cuff manometer and modified to maintain pressure in the range of 20–30 cmH_2_O, if necessary. Then, the BCP and air leak were checked while expanding the cuff in the same manner as in the supine position. After the BCPmax was measured, the BCP was lowered to the minimum value; the cuff manometer remained connected to the pilot balloon until the start of the operation. Two min after incision and insertion of the trocar for thoracoscopy, the BCP was measured again and whether the operative lung collapsed correctly in the operative field was checked by the operating surgeon. Then, the BCPmax was measured and maintained throughout the operation.

The baseline characteristics of the study population were collected perioperatively, including age, sex, height, weight, body mass index (BMI), diameter of the LMB, size of the used DLT, operative side (right or left), the angle at which the operating table was tilted, anesthesia time, operation time, study time (from anesthesia induction to measurement of the last BCP value) and average body temperature during the study period.

### 2.4. Study End Points

The primary outcome was the change in BCPi after the lateral decubitus positioning, that is the difference in BCP between the supine and lateral positions when injecting the BCVi of air. The secondary outcomes were the change in BCPi after starting the VATS operation; the changes in PIP, airway compliance (Cdyn = TVe/(PIP–PEEP)) and LMB length; the change in tracheal cuff pressure before and after the lateral positioning; and the relationships of variables such as airway pressure, compliance and BMI to the change in BCP after lateral positioning.

### 2.5. Sample Size

In a preliminary study, the difference in BCP when injecting the BCVi between the two positions was 2.3 cmH_2_O and the standard deviation was 6.53. Based on that result, we calculated that 66 patients would be required to achieve a power of 80% and a significance level of 5% (two-sided). Seventy-four patients were included to allow for a 10% dropout rate.

### 2.6. Statistical Analysis

The data were analyzed on an intention-to-treat basis and missing data were handled using the last observation carried forward method. According to the results of Kolmogorov–Smirnov test, normally and non-normally distributed data were presented as the mean (standard deviation) or the median (interquartile rage), respectively. Categorical data were presented as the number of patients (percentage).

The paired data at two time points such as the primary outcome were analyzed by a paired t-test or Wilcoxon signed-rank test. When analyzing by dividing into two groups, a repeated-measures analysis of variance (RM ANOVA) was used to compare BCPi at three time points. In the presence of interaction effects between time point and group variables, RM ANOVA was, respectively, performed for each group. The *p*-values for post-hoc multiple comparisons were adjusted using Bonferroni correction. When comparing variables between two groups, Student’s t-test or Mann–Whitney U test were used. Categorical data were compared using the χ^2^ test. Pearson’s correlations were calculated to determine the relationships between the change in BCP and other variables. A *p*-value < 0.05 was considered statistically significant. All statistical analyses were performed using IBM SPSS Statistics version 19.0.0 (IBM Corp., Armonk, NY, USA).

## 3. Results

**Figure 1:**
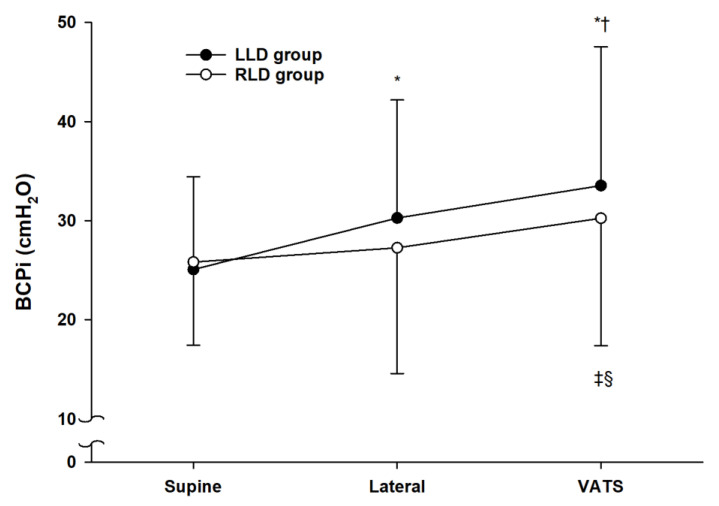
The changes of BCPi at supine position, after lateral positioning and after starting the VATS operation. The black circles show the mean BCPi values with standard deviation of the patients in the LLD group. The white circles show the mean BCPi values with standard deviation of the patients in the RLD group. * *p* < 0.001 compared to supine position, † *p* = 0.001 compared to lateral position, ‡ *p* < 0.05 compared to supine position, § *p* < 0.05 compared to lateral position. BCPi, initial established bronchial cuff pressure; VATS, video-assisted thoracoscopic surgery; LLD, left lateral decubitus; RLD, right lateral decubitus.

A total of 69 patients were included in the study. Patient characteristics are presented in Table 1. As the primary outcome, the BCPi increased from 25.4 ± 9.0 cmH_2_O in the supine position to 29.1 ± 12.2 cmH_2_O in the lateral decubitus position (*p* < 0.001) (Figure 1).

Out of the 69 participants, 43 patients underwent surgery in the left-lateral decubitus position (LLD group) and the remaining 26 patients in the right-lateral decubitus position (RLD group). The baseline characteristics were similar between the two groups (Table 1). The time course of the BCPi in the LLD group was significantly different from that of the RLD group (*p* = 0.043). In the LLD group, the BCPi increased significantly (*p* < 0.001) after lateral positioning and the beginning of surgery (Figure 1). In the RLD group, the BCPi did not show a significant increase before and after lateral positioning but showed a significant increase before and after starting the VATS operation (*p* = 0.033 compared to the supine position, *p* = 0.049 compared to the lateral position). The difference in value, such as ∆BCPi, from supine to lateral position was significantly higher in the LLD group than in the RLD group (mean ± SD, 5.2 ± 6.6 vs. 1.4 ± 7.2 with a mean difference of 3.8 and 95% CI of 0.3 to 7.2, *p* = 0.034); however, ∆BCPi from the lateral position to the start of the VATS operation showed no significant difference between the two groups (Table 2).

**Figure 2:**
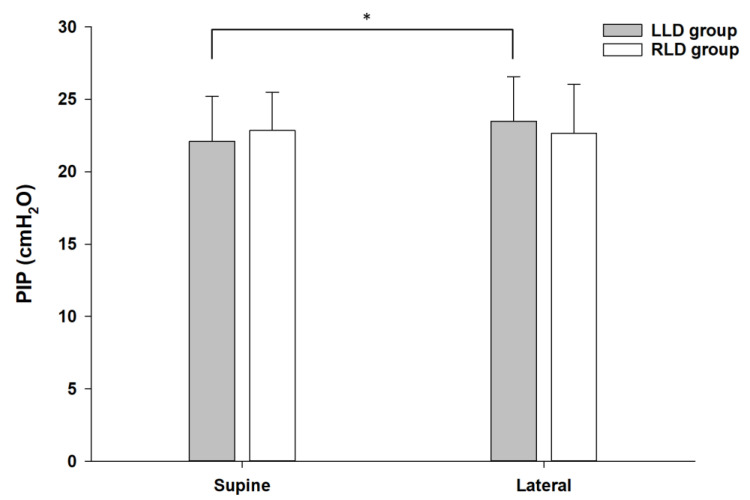
The changes of PIP at supine position and after later positioning. The grey boxes show the mean PIP with standard deviation of the patients in the LLD group. The white boxes show the mean PIP with standard deviation of the patients in the RLD group. * *p* = 0.001 compared to supine position. PIP, peak inspiratory pressure; LLD, left lateral decubitus; RLD, right lateral decubitus.

The PIP increased before and after lateral positioning from 22.4 ± 2.9 cmH_2_O to 23.2 ± 3.2 cmH_2_O (*p* = 0.010) in total patients, although Cdyn and LMB length did not significantly change. The PIP in the LLD group showed significant increase before and after positioning (*p* = 0.001), as in total participants; however, no such change was observed in the RLD group (Figure 2). The difference values, ∆PIP and ∆Cdyn, from supine to lateral position were significantly higher in the LLD group than in the RLD group (Table 2). ∆LMB tended to be lower in the LLD group than in the RLD group, although there was no significant difference. Regarding the relationship between the change in BCPi and the other variables, there was no correlation between patients of the LLD and RLD groups.

The tracheal cuff pressure increased from 27.8 ± 1.7 cmH_2_O in the supine position to 42.2 ± 11.9 cmH_2_O in the lateral position (*p* < 0.001). However, there was no significant difference in the tracheal cuff pressure at each time points or their difference values between the LLD and RLD groups (Table 2) and there was no correlation between the change in BCPi and tracheal cuff pressure.

## 4. Discussion

The present study showed that the initial established BCP of DLT increased by the positional change from supine to lateral decubitus. The changes in BCPi between the supine position and the beginning of surgery were different between LLD and RLD positions. The difference value of BCPi, denoted as ∆BCPi, from supine to lateral position was significantly higher in LLD than in RLD, although ∆BCPi from lateral position to starting of the VATS operation showed no significant difference between the two positions. Taken together, these results suggested that an increase in BCP was more attributed to lateral positional change than surgical manipulation, especially in LLD position.

A previous study related to the postural change (e.g., head-down tilt) explained that gravity-induced morphological and conformational change of the trachea could affect the cuff pressure of the ETT [3]. Likewise, it is presumed that this gravity-induced mechanism also works during the postural change from supine to lateral position. Moreover, a recent study showed that the LMB deviated towards the cranial side when changing from supine to LLD position [20]. The angle formed by the LMB and tracheal axis increased in LLD compared with that in the supine position and even the increase on the distal side of the LMB showed significant change. In contrast, the curvature of LMB was reduced in RLD compared with that in the supine position [20]. The formation of such curvature has been attributed to the influence of adjacent organs, such as elevation of left ventricular end-diastolic pressure, dilation of the left atrium, or sandwiching between the left atrium and aortic arch [21,22]. After the postural change, not only the curvature, but also the length of the LMB can be altered. In the present study, the change in LMB length during lateral positioning tended to be smaller in LLD than in RLD, albeit small and statistically insignificant. In addition to the gravitational effect, these LMB’s anatomical changes related to the curvature and length can be an additional factor in increasing the pressure of the bronchial cuff placed within the LMB after changing from the supine to lateral position and the effect was more pronounced in LLD than in RLD. In the RLD position, since the LMB and bronchial cuff of the DLT are located in a non-dependent portion, they could be relatively less affected by gravitational effects.

In addition to the lateral positional change, the effect of surgical procedures on the BCP after the insertion of the port should be considered. Wu et al. demonstrated that the CO_2_ insufflation itself, even without the postural change, caused an increase in the cuff pressure of ETT in patients who underwent laparoscopic surgery [3]. Yamada et al. revealed that the required sealing BCV decreased after opening the chest in thoracic surgery [23] and that result suggested that using the initial volume of air could cause over-inflation of the bronchial cuff after thoracotomy. However, unlike laparoscopic surgery, the VATS operation used in the present study does not generally require CO_2_ [24] due to the inherent rigidity of the thoracic cage. In addition, the present study included patients who did not require open thoracotomy. We believe these factors are the reason why ∆BCPi from supine to lateral was greater than that from lateral position to starting of the VATS operation, albeit not significantly different in LLD group; therefore, the change in surgical condition had less influence on the change in BCP compared with the postural change in LLD position.

The increase in PIP was significantly greater in LLD than in RLD probably because the relatively larger right lung is located above the left lung and LMB [25], thereby exerting a gravitational effect. Moreover, the diameter of the right bronchus is smaller than that of the left side [26] and the cranial deviation of the LMB is larger than that of the right side in lateral position [20]; hence, these factors may also have contributed to these results. In order to investigate the effect of PIP on the BCP, it was necessary to analyze only the results of the LLD group who had OLV of the left lung where the bronchial cuff was located. The present study showed that both BCP and PIP were significantly increased from supine to LLD position, but there was no significant correlation between the BCP and PIP. The PIP has been reported to be associated with changes in cuff pressure of the ETT in certain conditions. In obese patients with BMI over 30 kg/m^2^ who underwent laparoscopic pelvic surgery requiring the Trendelenburg position, i.e., in a model of acute decrease in respiratory compliance, increased airway pressure was associated with the cuff pressure of the ETT [27], which was explained as the elevation in the airway pressure could be transmitted to the ETT cuff. Since most participants of our study were non-obese, it was likely that similar results could not be obtained.

Similar to the results in a study using single-lumen ETT [14], in the present study, the tracheal cuff pressure also showed a significant increase by posture change from the supine to lateral position. According to the study by Kim et al. [14], the tracheal cuff pressure increased from 20 cmH_2_O to 31 ± 7 cmH_2_O in tapered-shaped cuffs and 25 ± 4 cmH_2_O in cylindrical-shaped cuffs. The increase in the tracheal cuff of the DLT was greater in the present study. Similar results were reported in the study of Kim et al. [28], which compared the increase in the tracheal cuff pressure of single-lumen ETT and DLT during the TEE probe insertion. The increase in tracheal cuff was greater in patients who received a DLT than in those receiving a single-lumen ETT and the authors explained that it was attributed to the large external diameter of the DLT. Furthermore, its non-compressible portion might have already occupied the same limited tracheal space than in single-lumen ETT. Therefore, when using DLT, it is necessary to monitor the tracheal cuff pressure as well as the BCP during a positional change.

There are some limitations to this study. First, equilibration of the intracuff pressure and measurement of the cuff pressure using cuff manometer while deflating and inflating manually could inevitably result in slight measurement errors. The measurement of LMB length using FOB also could result in such errors, albeit minimal. The manipulation of the cuff manometer and FOB was performed by skilled clinicians with experience. Second, the cuff pressure and other variables were observed only until the start of surgery and subsequent time points including the postoperative period were excluded from the study, since there were large differences in the operation time of VATS at our institution. Prolonged surgery with considerable surgical manipulation of the lungs or bronchi can lead to airway edema or mucus hypersecretion and influence the cuff pressure or other respiratory variables. If the limitation related to the operation time can be resolved in the future, further studies are required on the postoperative outcomes such as relationship between BCP and postoperative sore throat or airway injuries. Finally, the sample size was originally calculated for the primary outcome, i.e., the change in BCP before and after lateral positioning; therefore, it might be insufficient to perform analyses of secondary outcomes. In particular, in order to investigate the effect of PIP on the BCP, only the PIP data of the LLD group were used and, if only a group of patients with added other specific conditions (e.g., high BMI) is to be analyzed, it is necessary to perform the further studies by calculating the sample size for patients with such conditions.

## 5. Conclusions

In conclusion, positional change from supine to lateral decubitus could increase the initial established BCP of DLT and the increase was significantly greater in LLD that in RLD, which was primarily due to gravitational effects. This BCP also increased after the beginning of the VATS in both LLD and RLD positions. Therefore, clinicians should be aware that the BCP may increase when performing lateral positioning, particularly to the LLD position, and when proceeding with the surgery. The routine and continuous monitoring of the BCP should be considered if possible when using the DLT in thoracic surgery.

## Figures and Tables

**Table 1:** Characteristics of patients before and during study period.

Variable	All Patients	LLD Group	RLD Group	*p*-Value
	(*n* = 69)	(*n* = 43)	(*n* = 26)	
Age (years)	53 (21–66)	55 (36–66)	48 (19–66)	0.373
Sex (male/female)	55/14	34/9	21/5	1.000
Weight (kg)	61.9 ± 9.5	61.4 ± 9.5	61.9 ± 9.5	0.661
Height (cm)	168.3 ± 9.3	168.3 ± 9.3	168.3 ± 9.3	0.431
BMI (kg m^−2^)	21.9 ± 3.5	21.6 ± 3.1	22.5 ± 3.9	0.323
Diameter of LMB (mm)	11.3 ± 1.3	11.2 ± 1.3	11.4 ± 1.2	0.403
Tube size (37 Fr/35 Fr)	52/17	31/12	21/5	0.567
Table inclination angle of head side (°)	16.8 ± 5.7	16.4 ± 4.9	17.5 ± 6.7	0.511
Anaesthesia time (min)	154.0 (118.5–252.5)	154.0 (117.0–256.0)	150.5 (119.5–259.8)	0.892
Operation time (min)	90.0 (53.0–152.5)	92.0 (54.0–148.0)	88.5 (52.5–172.5)	0.921
Study time (min)	53.2 ± 16.2	54.0 ± 17.6	51.9 ± 13.8	0.601
Body temperature (°C)	36.0 ± 0.3	36.0 ± 0.3	36.1 ± 0.3	0.226
Number of patients whose BCPi > 40 cm H_2_O after lateral positioning	10	7	3	0.732

Data are presented as median (IQR) number or mean ± SD. LLD, left lateral decubitus; RLD, right lateral decubitus; BMI, Body mass index; LMB, left main bronchus; BCPi, initial established bronchial cuff pressure.

**Table 2:** Characteristics of patients, anesthesia and surgery.

Variable	LLD Group	RLD Group	*p*-Value
	(*n* = 43)	(*n* = 26)	
BCPi (cmH_2_O)			
∆ (from supine to lateral)	5.2 ± 6.6	1.4 ± 7.2	0.034
∆ (from lateral to VATS)	3.3 ± 5.5	2.9 ± 5.9	0.842
Supine position	25.1 ± 9.4	25.8 ± 8.4	0.736
Lateral position	30.3 ± 11.9	27.3 ± 12.7	0.324
VATS operation	33.5 ± 14.0	30.2 ± 12.9	0.333
PIP (cmH_2_O)			
∆ (from supine to lateral)	1.0 (0–3.0)	−1.0 (−1.0–0)	0.001
Supine position	22.1 ± 3.1	22.8 ± 2.6	0.305
Lateral position	23.5 ± 3.1	22.7 ± 3.4	0.295
Cdyn (ml/cmH_2_O)			
∆ (from supine to lateral)	−0.9 (−3.3–0.4)	1.3 (−1.3–2.5)	0.004
Supine position	23.6 ± 5.5	22.4 ± 3.1	0.250
Lateral position	22.1 ± 4.2	22.6 ± 5.7	0.664
LMB length (mm)			
∆ (from supine to lateral)	−0.1 ± 0.5	0.2 ± 0.6	0.074
Supine position	5.0 (4.7–5.6)	4.9 (4.1–5.3)	0.122
Lateral position	5.0 (4.6–5.5)	4.9 (4.5–5.6)	0.583
Tracheal Cuff Pressure (cmH_2_O)			
∆ (from supine to lateral)	15.0 ± 13.9	13.5 ± 8.8	0.691
Supine position	27.7 ± 1.8	28.0 ± 1.6	0.537
Lateral position	42.7 ± 13.7	41.5 ± 8.7	0.753

Data are presented as median (IQR), number or mean ± SD. LLD, left lateral decubitus; RLD, right lateral decubitus; BCPi, initial established bronchial cuff pressure; VATS, video-assisted thoracoscopic surgery; PIP, peak inspiratory pressure; Cdyn, airway compliance; LMB, Left main bronchus.

## Data Availability

The data presented in this study are available on request from the corresponding author.
